# Exploring heterogeneous expression of beta-actin (*ACTB*) in bladder cancer by producing a monoclonal antibody 6D6

**DOI:** 10.1186/s12894-024-01489-6

**Published:** 2024-06-12

**Authors:** Mohammadrasul Zareinejad, Zahra Faghih, Amin Ramezani, Akbar Safaei, Abbas Ghaderi

**Affiliations:** 1 Shiraz Institute for Cancer Research, School of Medicine, Shiraz University of Medical Sciences, Shiraz, Iran; 2 Department of Immunology, School of Medicine, Shiraz University of Medical Sciences, Shiraz, Iran; 3 Department of Medical Biotechnology, School of Advanced Medical Sciences and Technologies, Shiraz University of Medical Sciences, Shiraz, Iran; 4 Department of Pathology, School of Medicine, Shiraz University of Medical Sciences, Shiraz, Iran

**Keywords:** Bladder cancer, Hybridoma, Monoclonal antibody, Biomarkers, Beta-actin, *ACTB*

## Abstract

**Background:**

To predict outcomes and identify potential therapeutic targets for cancers, it is critical to find novel specific biomarkers. The objective of this study was to search for and explore novel bladder cancer-associated protein biomarkers.

**Methods:**

A library of monoclonal antibodies (mAbs) against the JAM-ICR cell line was first generated, and clones with high affinity were selected. Hybridomas were screened using bladder cancer (BLCA) cell lines and normal cells. The target of the selected mAb was then characterized through immunoaffinity purification, western blotting, and mass spectrometry analysis. Expression of the target antigen was assessed by flow cytometry and IHC methods. Several databases were also used to evaluate the target antigen in BLCA and other types of cancers.

**Results:**

Based on screenings, a 6D6 clone was selected that recognized an isoform of beta-actin (*ACTB*). Our data showed that *ACTB* expression on different cell lines was heterogeneous and varied significantly from low to high intensity. 6D6 bound strongly to epithelial cells while showing weak to no reactivity to stromal, endothelial, and smooth muscle cells. There was no association between *ACTB* intensity and related prognostic factors in BLCA. In silico evaluations revealed a significant correlation between *ACTB* and overexpressed genes and biomarkers in BLCA. Additionally, the differential expression of *ACTB* in tumor and healthy tissue as well as its correlation with survival time in a number of cancers were shown.

**Conclusions:**

The heterogeneous expression of *ACTB* may suggest the potential value of this marker in the diagnosis or prognosis of cancer.

**Supplementary Information:**

The online version contains supplementary material available at 10.1186/s12894-024-01489-6.

## Background

Bladder cancer (BLCA), also known as urinary bladder cancer, is the 10th most prevalent cancer with an increasing prevalence, particularly in developed countries. With approximately 200,000 mortalities in 2020, it is the 12th most dangerous malignancy, accounting for 2.1% of all cancer-related deaths [1]. Despite an increase in incidence, BLCA mortality has significantly decreased globally [2]. Men are four times more susceptible than women to get BLCA, making it the sixth most prevalent and ninth most lethal neoplasm in men [3]. The 5-year survival rate for BLCA in the US is 77.1%, but it varies based on the time of diagnosis and the spread of tumor cells [4].

The first-line treatment for advanced and metastatic BLCA is chemotherapy, but due to the dismal objective response rate, the 5-year survival rate is low. Resistance to chemotherapeutic medicines severely restricts the efficacy of current standards in BLCA [5]. Targeted therapies, as another strategy, are frequently employed and exhibit positive therapeutic outcomes in a variety of malignancies, including colon, lung, and breast cancers [5, 6]. Although effective candidates for BLCA biomarkers have not yet been introduced, molecular diagnostics play an important role in managing the disease. Therefore, it is essential to identify novel particular biomarkers that can be used as therapeutic and prognostic targets [7].

**Fig. 1:**
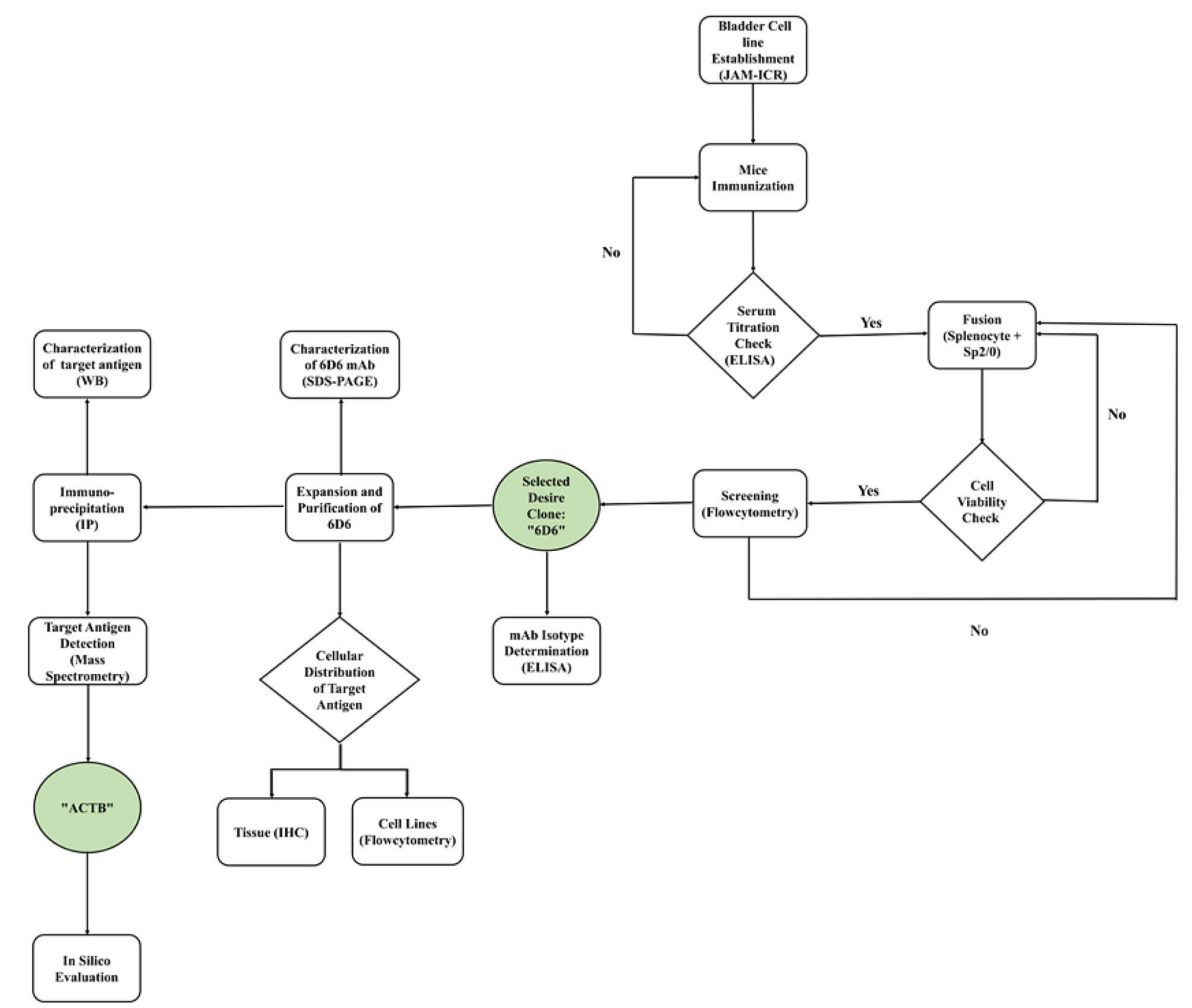
Workflow diagram. WB: western blot, IHC: Immunohistochemistry

New biomarkers have been discovered using a variety of techniques, including genomics, metabolomics, proteomics, and monoclonal antibody (mAb)-based technologies [8, 9]. The mAb-based strategy generates a panel of highly reactive monoclonal antibodies against tumor tissue that recognizes various epitopes. New targets with conformational epitopes or posttranslational modifications can be discovered with mAb-based strategies that cannot be detected by genomic or proteomic approaches alone [10]. Additionally, these techniques can provide useful details regarding molecular interactions and antigen localization [11, 12]. To distinguish tumor-specific antigens in their native forms, using the whole cell as an immunogen is an ideal option [10]. Current commercial cell lines no longer resemble the original tumors because they have undergone numerous passages and lost their characteristics. In light of this, the JAM-ICR cell, a new BLCA cell line isolated from bladder carcinoma specimens in our laboratory [13], was employed as an antigen source. Thus, the aim of this study was to produce a mAb library against JAM-ICR using the hybridoma approach, screen them against normal cells, and select a suitable mAb clone. After isotype determination and assessing the reactivity against various cancer cell lines and tumor samples, the target antigen was identified and evaluated (workflow diagram is depicted; Fig. 1).

## Methods

### Cell culture

Several cell lines (5637, MRC-5, MDA-MB-231, MCF-7, U87, SW1116, Jurkat, HT29, EJ [MGH-U1], AGS, Raji, and HEK293) were obtained from the National Cell Bank (Pasture Institute, Iran). JAM-ICR, a new cell line isolated from a 64-year-old patient with high-grade (grade IV) BLCA, was established at the Shiraz Institute for Cancer Research [13]. Adipose-derived mesenchymal stem cells (MSCs) were derived from breast adipose tissues of a healthy donor who underwent cosmetic mammoplasty by the explant method. Cell lines were cultured in RPMI-1640 medium (Gibco, USA) supplemented with 10% fetal bovine serum (FBS; Gibco) and 1% penicillin‒streptomycin (P/S; Sigma‒Aldrich, Germany). HEK293 and MSCs were cultured in Dulbecco’s modified Eagle medium (DMEM). The selection medium was composed of RPMI-1640 supplemented with hypoxanthine-aminopterin-thymidine (HAT) medium (2X, Sigma‒Aldrich), 20% FBS, nonessential amino acids (Gibco), 1 mM sodium pyruvate (Gibco), and conditioned medium obtained from cultured peritoneal feeder cells. HAT medium was replaced with hypoxanthine-thymidine (HT) medium (Sigma‒Aldrich) and complete culture medium on the 14th and 21st days of fusion, respectively. All cells were grown in a humidified 5% CO_2_ incubator at 37 °C.

### Immunization of BALB/c mice with the JAM-ICR cell line

Inbred 6-week-old BALB/c mice were obtained from the Pasteur Institute of Iran (Tehran, Iran). When the JAM-ICR cell line reached the logarithmic phase of growth, the cells were scraped and collected. Then, 500 µl of cell suspension with a density of 1 × 10^7^ cells/ml was adjusted and injected intraperitoneally. The subsequent boosts were performed at three-week intervals and repeated until a sufficient serum titer was obtained. The last boost was injected three days before the fusion.

### ELISA

An indirect ELISA was used to titrate the concentration of antibodies in the mouse serum. Blood samples from the tail veins of inoculated mice were taken one day before and one week after each injection. JAM-ICR cell lysate was obtained by RIPA lysis buffer and diluted in carbonate/bicarbonate buffer (5 µg/well: Merck, Germany). Then, 100 µL JAM-ICR lysate was coated in MaxiSorp flat-bottom 96-well plates (Nunc, Denmark). After overnight incubation, 250 µL blocking buffer [1% bovine serum albumin (BSA); Biosera, France] was added and incubated at room temperature (RT) for 2 h. Following the addition of mouse sera (1:500) to wells and washing, 100 µl of HRP-conjugated goat anti-mouse antibody (1:1500, BD Biosciences, USA) was incubated for 1 h at 37 °C. Then, 100 µl tetramethylbenzidine (TMB) substrate solution (Invitrogen, USA) was added for 15 min at RT, and the reaction was stopped with 100 µl H_2_SO_4_ (0.16 M). The optical density was measured at 450 nm using a microplate reader (Anthos 2020, Austria). All washes were performed with 1X PBS buffer containing 0.05% Tween-20 (Bio-Rad, USA).

### Production of hybridoma

The fusion procedure was done according to previous work in our laboratory [14, 15]. Briefly, splenocytes and SP2/0-Ag14 cells at a 5:1 ratio in serum-free RPMI-1640 were mixed and centrifuged at 300×g for 5 min. Then, 1000 µl PEG (Sigma‒Aldrich) was added to the cell pellet (12 × 10^7^) dropwise over 1 min while agitating the tube. The fusion mixture was incubated for an additional 3 min, and then 10 ml of serum-free RPMI-1640 was added over the course of 4 min. The cell suspension was centrifuged and then adjusted to 1.5 × 10^6^ cells/ml in the selection medium.

### Flow cytometry

The surface expression of the target antigen was evaluated with flow cytometry [16]. For this purpose, 100 µl of supernatant from hybridoma cells was added to cell suspensions (2 × 10^5^ cells/tube) and incubated for 60 min at 4 °C. Following washing cells with 2 ml staining buffer, the cells were incubated for 30 min at 4 °C with 50 µl diluted FITC-conjugated sheep anti-mouse Ig secondary antibody (1 µl antibody in 49 µl staining buffer; SINA BIOTECH, Iran). After washing, data were acquired on a 4-color flow cytometer instrument (BD Biosciences) and analyzed by FlowJo software (version X.0.7, USA). Each test was done three times separately, and the frequencies of positive cells are shown.

### Isotype determination of mAb

A limiting assay was performed three times to isolate single clone-producing antibodies. The isotype of antibodies was then evaluated with an eBioscience™ Mouse Ig Isotyping ELISA Kit (Invitrogen, Austria) according to the manufacturer’s instructions.

### Expansion and purification of mAb

Female BALB/c mice (6 weeks old) were primed by intraperitoneal injection of 500 µl/mouse Pristane (Sigma‒Aldrich). After 7 days, hybridoma cells were harvested, and 500 µl of cell suspension (7 × 10^6^ cells/ml) was administered. The ascitic fluid was collected and purified by a Hi-Trap protein G column (GE Healthcare, Sweden) and fast protein liquid chromatography (FPLC) instrument (GE Healthcare). The sample was diluted with binding buffer (20 mM phosphate) and applied to the column. Then, the column was washed with binding buffer to remove the unbound proteins until the absorbance reached a steady baseline of 0.1 milliabsorbance unit (mAU). Attached antibodies were eluted with elution buffer (100 mM glycine, pH = 2.7) at a flow rate of 1 ml/min. Following dialysis with 1X PBS, the concentration of the mAb was determined using a NanoDrop 2000c Spectrophotometer (Thermo Fisher Scientific, USA).

### Western blot

Purified mAb (20 µg) and target antigen (40 µg) were loaded on a 12.5% polyacrylamide gel under reduced conditions. The gel was either transferred to a PVDF membrane or stained with colloidal Coomassie Brilliant Blue G-250 (Bio-Rad). The transfer was done using 25 constant voltage and 2.5 limited ampers for 80 min. The blot was placed in blocking buffer (3% BSA in PBS containing 0.15% Tween-20) for 2 h, and then 20 µg/ml primary antibodies were added and incubated at RT with shaking for 1 h. After washing, the blot was incubated for 1 h at RT with HRP-conjugated goat anti-mouse Ig (BD Biosciences, 1:3000 in blocking buffer). After soaking the PVDF membrane in enhanced chemiluminescence substrate (Bio-Rad) for 5 min in the dark, the protein was detected using a ChemiDoc imaging system (Bio-Rad).

### Immunoprecipitation (IP)

Target antigen was isolated with a Pierce Crosslink Immunoprecipitation Kit (Thermo Fisher Scientific). According to the company’s instructions, the lysate of JAM-ICR was obtained by using cold IP lysis buffer and coincubated with protein A/G agarose. After cross-linking the selected antibody to agarose with disuccinimidyl suberate (DSS), unbound proteins were washed, and the target protein was collected by elution buffer. The output was verified by western blotting, and the specific target was excised from the polyacrylamide gel and analyzed by liquid chromatography with mass spectrometry (LC/MS). Accordingly, the beta-actin (*ACTB*) protein was introduced as the target antigen of 6D6.

### Immunohistochemistry (IHC)

Expression of the target antigen in different bladder tumor tissues was assessed by immunohistochemical staining (IHC). In this connection, resected specimens from 35 patients (29 males and 6 females) with a mean age of 66 ± 2 years old were collected. Five samples from von Brunn’s nests (proliferating epithelial cells; benign tumors) were also assessed. Furthermore, nearby normal tissues were used for benign BLCA situations. The IHC procedure was carried out in accordance with our lab’s setup [13]. The intensity of expression was qualified by scoring from 0 to 2 (0: negative, 1: low, 2: high). The relationship between *ACTB* intensity and prognostic factors, including T- and N-stages, histological grade, tumor necrosis, lymph node involvement, carcinoma in situ, perivesical fat, invasion of the tumor to the adjacent muscle, perineural, lamina propria, and lymphovascular invasion, was also evaluated.

### In silico evaluation

Gene Expression Profiling Interactive Analysis-2 (http://gepia2.cancer-pku.cn/), STRING (https://string-db.org/), cBioPortal (cBioPortal for Cancer Genomics, http://www.cbioportal.org), Catalog of Somatic Mutations in Cancer (COSMIC) database (https://cancer.sanger.ac.uk/cosmic), and the University of California Santa Cruz (UCSC) Genome Browser (https://genome.ucsc.edu/) web servers were used to study *ACTB* in BLCA and other cancers. First, the expression of *ACTB* in tumor and normal tissue and then in different stages and survival were obtained in BLCA. Then, the correlation between *ACTB* and the overexpressed gene and BLCA tumor biomarkers [17] was evaluated. Furthermore, the GEPIA2 server recommended 10 genes that have similar expression patterns with *ACTB* in BLCA. The expression, survival, and interaction of *ACTB* were evaluated in various cancers. The interaction network of *ACTB* with other proteins and genes was obtained from STRING and UCSC servers, respectively.

### Statistical analysis

SPSS (version 25.0, USA) and GraphPad Prism (version 6.01, USA) software were used to conduct the statistical analysis and depict figures, respectively. The expression of the target antigen in different cell lines were compared using Mann-Whitney U test. The relationship between the intensity of *ACTB* and prognostic factors was analyzed with the chi-square (*x*^2^) test. Additionally, the survival of patients with different expression levels of *ACTB* was compared with the Kaplan–Meier estimator and the log-rank test. A P-value of less than 0.05 was regarded as statistically significant, and the data are reported as the mean ± SD.

## Results

### Production and screening of mAbs against JAM-ICR

**Fig. 2:**
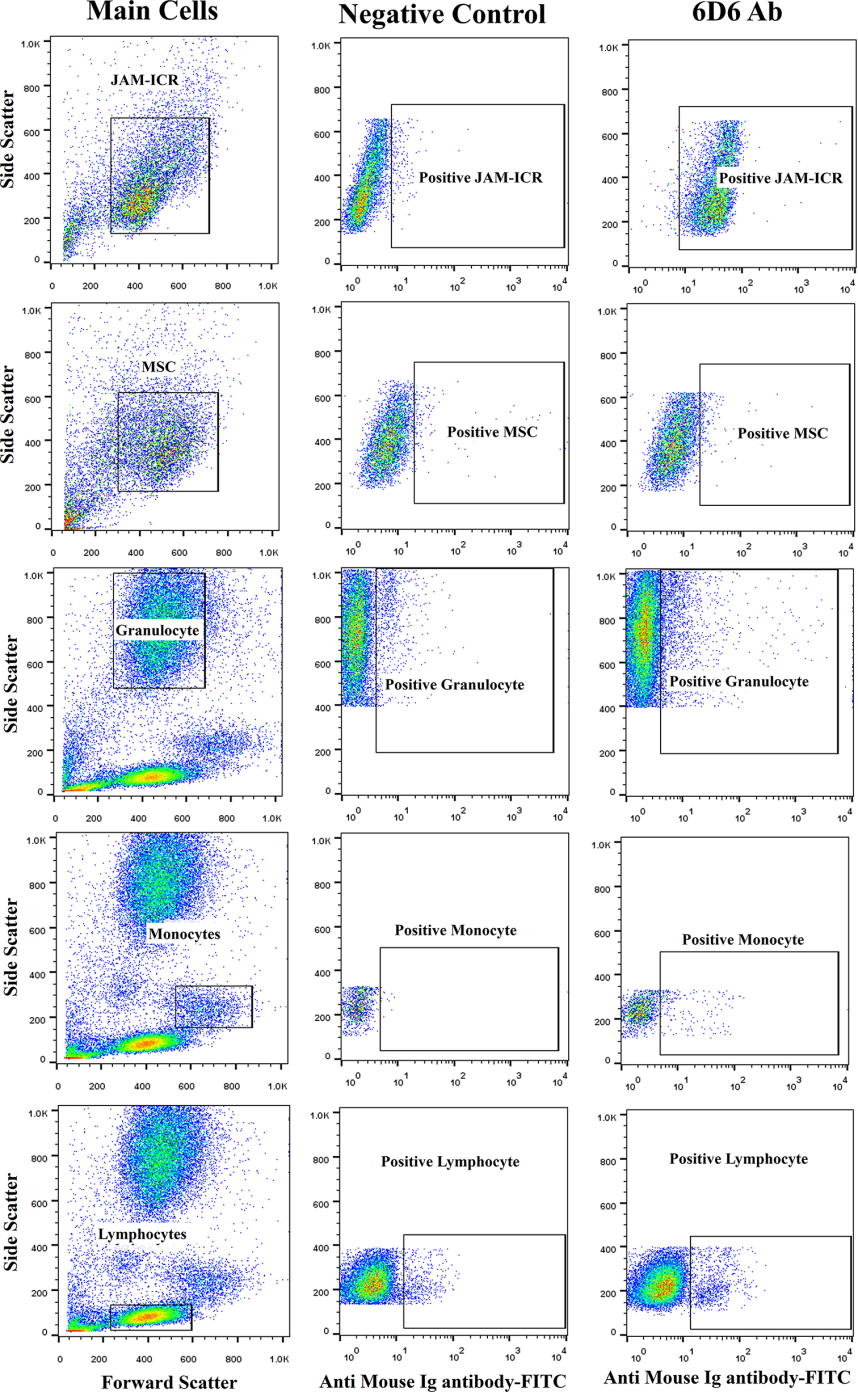
Reactivity of 6D6 mAb with JAM-ICR, MSCs, granulocytes, monocytes, and lymphocytes with flow cytometry. The main cells were selected based on their size and granularity (FSC/SSC), and then positive cells (positive FITC) were gated. Negative control cells were treated with only the anti-mouse Ig secondary antibody. MSC: Adipose-derived mesenchymal stem cells

The supernatant of hybridoma cells was assessed by flow cytometry, and the reactive clones with JAM-ICR were selected for the second screening. The reactivity of selected clones with human leukocytes and MSCs from breast tissue (as normal cells) was also assessed. Of them, one hybridoma clone (namely, 6D6) displayed high reactivity to JAM-ICR but low reactivity to human MSCs of normal breast tissue (3.1%) and leukocytes (3.1% lymphocytes, 9.6% granulocytes, and 9.7% monocytes; Fig. 2). After limiting dilution, the isotype and light chain of 6D6 were determined to be IgG1 with a kappa light chain.

### Purification of mAb

**Fig. 3:**
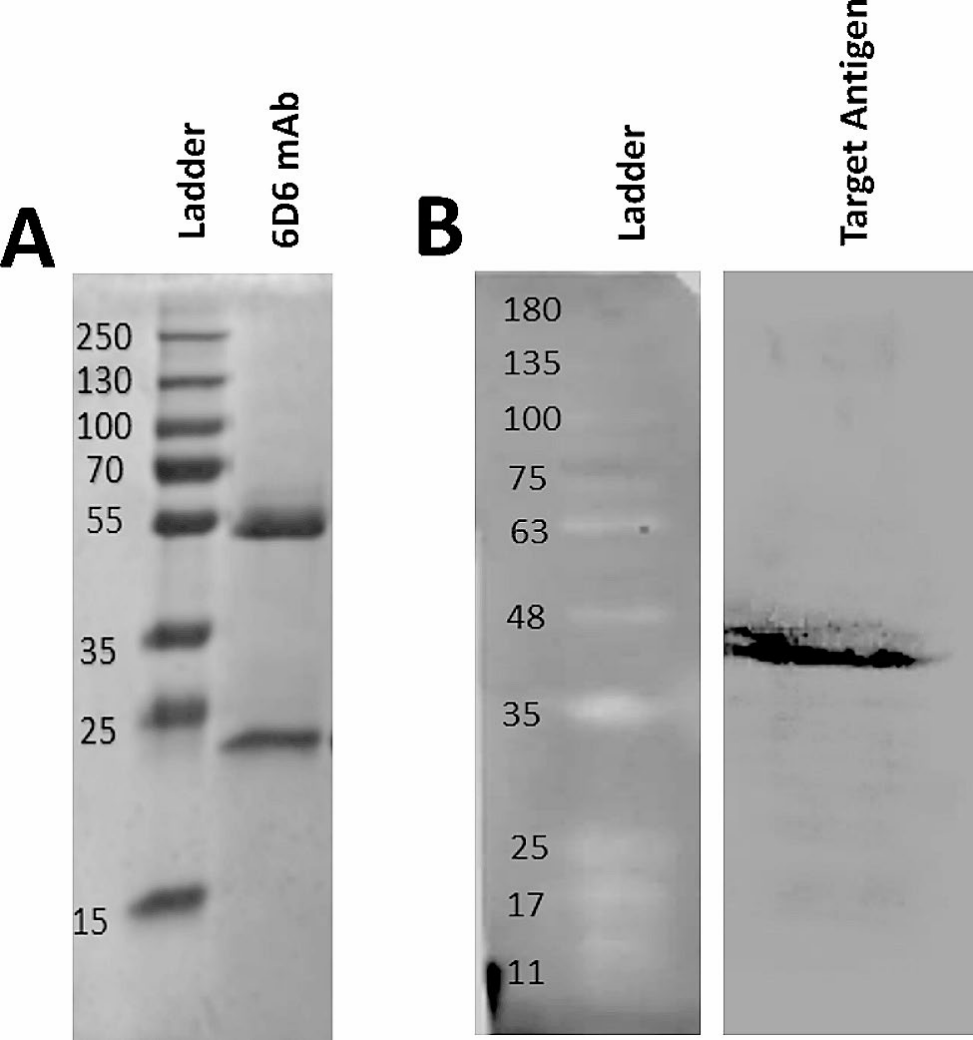
Characterization of the purified mAb and the target antigen. SDS‒PAGE electrophoresis of the purified monoclonal antibody 6D6 under reducing conditions showed two bands at 50 and 25 kDa (**A**). After purification with the immunoprecipitation strategy, the target antigen of 6D6 was run on SDS‒PAGE and then transferred to a PVDF membrane for western blotting (**B**). Full-length of the gel (Figure S1 with marker) and the original blot (Figure S2 with marker) are presented in supplementary files. Additionally, Fig. 3B with different contrast (using the Image Lab software setting) was also added to the supplementary file as Figure S3

Following the injection of antibodies into the peritoneum of mice, ascitic fluid was collected and purified with FPLC. The purity process was checked by SDS‒PAGE under reducing conditions. As shown in Fig. 3A, antibody components, including heavy chain (50 kD) and light chain (25 kD), were successfully separated.

### Identification of target antigens

Four processes were performed to identify the target antigen: IP, SDS‒PAGE, western blotting, and mass spectrometry analysis. First, the bound target antigen was separated with an immunoprecipitation assay and then applied to the western blotting (Fig. 3B). After confirmation by western blotting, the target antigen was excised from the gel and identified by mass spectrometry (LC/MS). Analysis of MS revealed that the target antigen of 6D6 is the *ACTB* protein (Table 1).

**Table 1:** Identification of 6d6 target antigen by LCMS/MS

Clones	Protein Name	UniPort accession No.	Unused	Peptides (95%)	Coverages%
**6D6**	*ACTB* (actin-beta)	P60709	78.26	189	81.4%

### Reactivity of the 6D6 antibody with cancer cell lines

**Fig. 4:**
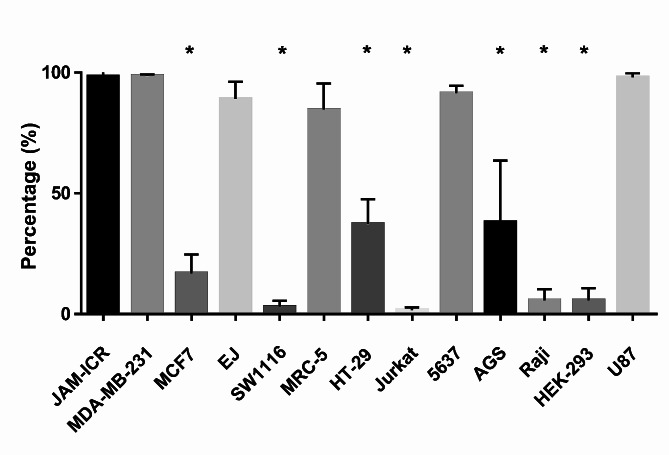
Evaluation of 6D6 reactivity with different cell lines by flow cytometry. The expression of *ACTB* on different cell lines was heterogeneous and varied from low (MCF-7, SW1116, Jurkat, Raji, and Heck293) to moderate (HT-29, AGS) and high (5637, MRC5, MDA-MB-231, U87, and EJ). Each test was performed three times separately, and the frequencies of each cells were compared with JAM-ICR. * = P-value < 0.05

Flow cytometry was applied to evaluate the expression of target antigens on the surface of other cancer cell lines, including 5637, MIA-PaCa-2, MRC-5, MDA-MB-231, MCF-7, U87, SW 1116, Jurkat, HT29, EJ (MGH-U1), AGS, Raji, and HEK293 cells. As shown in Fig. 4, the expression of *ACTB* on different cell lines was heterogeneous and varied from low (MCF-7, SW1116, Jurkat, Raji, and Heck293) to moderate (HT-29, AGS) and high (5637, MRC5, MDA-MB-231, U87, and EJ). This difference in the expression of *ACTB* was significant between the JAM-ICR with MCF-7, SW1116, Jurkat, Raji, Heck293, HT-29 and AGS.

### Cellular distribution of *ACTB*

**Fig. 5:**
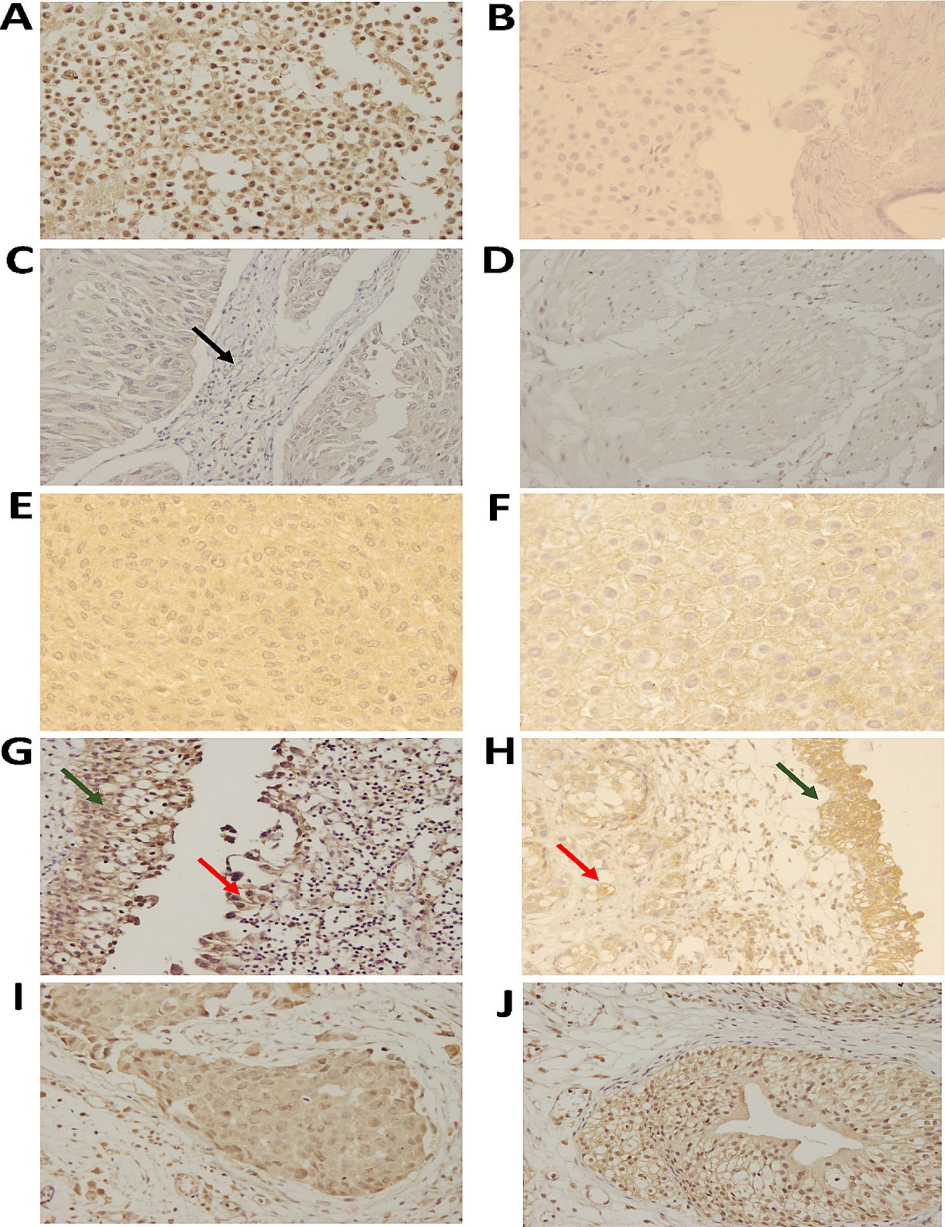
Cellular distribution of *ACTB* expression in bladder tissue. JAM-ICR cells were stained as a positive control (**A**), and a sample without 6D6 was considered a negative control (**B**). 6D6 showed no or low reactivity with stromal cells (**C**) and smooth muscle cells (**D**). Both cytoplasmic (**E**) and membranous (**F**) patterns of *ACTB* were observed. *ACTB* expression was compared between tumor tissue (red arrow) and normal adjacent tissue (green arrow) (**G**-**H**) and high stage (**I**) and low stage (**J**) BLCA

**Fig. 6:**
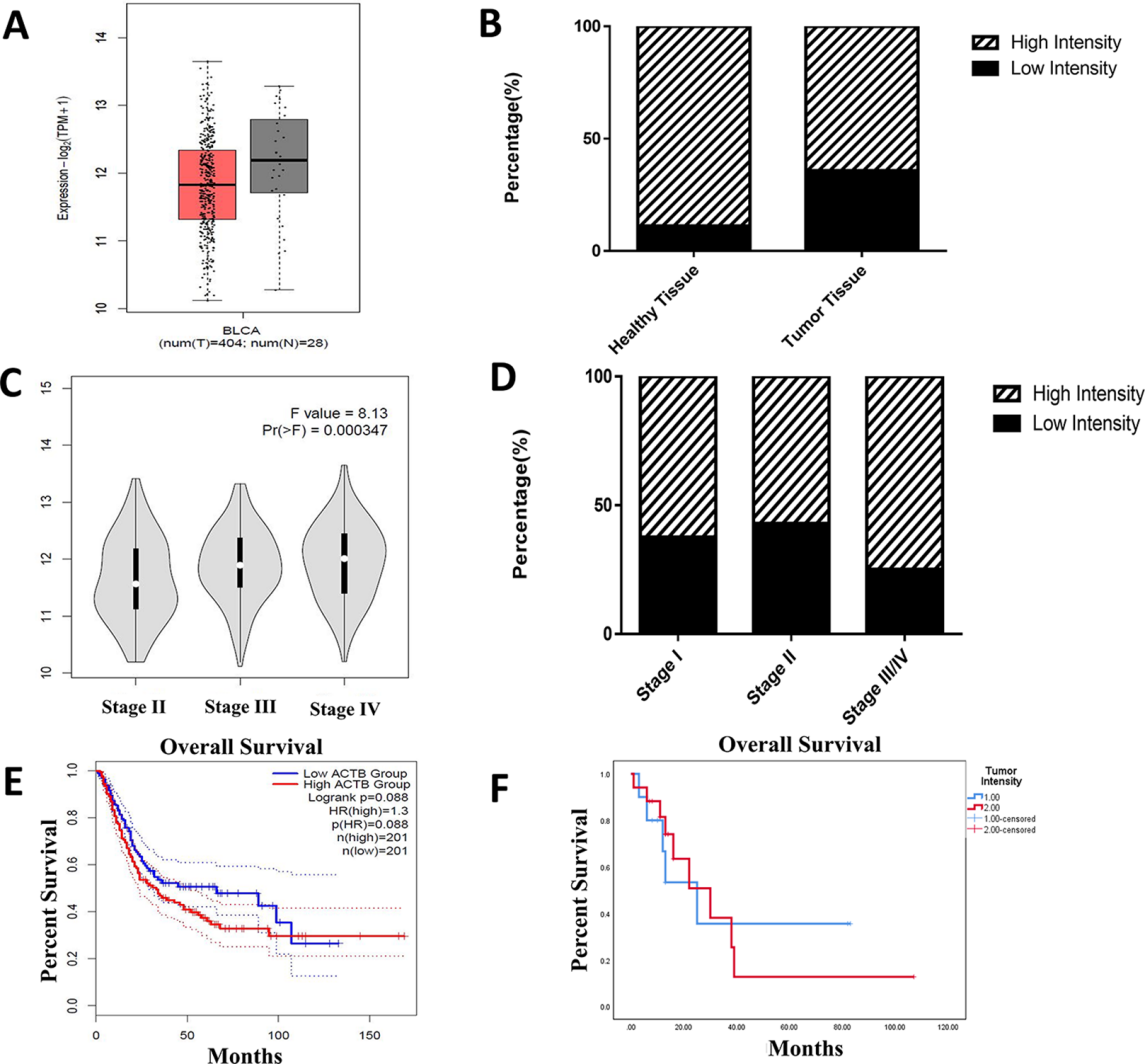
The in silico (**A**, **C**, **E**) and in vitro (**B**, **D**, **F**) evaluation of *ACTB* expression in bladder cancer. *ACTB* expression was not significantly different between tumor tissue and adjacent normal tissue (**A**, **B**). However, an in silico study showed a significant increase in *ACTB* expression in stage IV (**C**), and our IHC results did not show a significant difference (**D**). The survival time in patients with high *ACTB* expression was not different from that in patients with low *ACTB* expression (**E**, **F**). In silico data were obtained from the GEPIA2 database. Although the GEPIA2 database is mainly concerned with the analysis of mRNA expression levels in different tissues, the employed mAb-based approach enables the detection of differential biomarkers at the protein level

The binding of the 6D6 antibody to epithelial and other cells in tissue was investigated. The results indicated that this antibody was bound to all epithelial cells in tumor tissue and adjacent normal epithelial cells (100%; Fig. 5). High expression of *ACTB* was seen in 88.9% and 64.3% of adjacent normal epithelial cells and tumor tissue, respectively; however, this difference was not significant (Fig. 6B). A similar expression level was also observed between benign tumor tissue (von Brunn nests) and malignant tumor tissue. Additionally, no difference was found between stages (Fig. 6D). Among 35 samples, the expression pattern of *ACTB* was cytoplasmic, while in 16 samples (45.7%), membranous expression was also seen. High expression of *ACTB* was observed in 66.7% of cells with a membranous pattern, but this difference was not significant. Despite the lack of reactivity to stromal cells (SCs), *ACTB* was expressed with low intensity on 42.1% of smooth muscle cells (SMCs), 55.9% of endothelial cells, and 51.5% of lymphocytes. There was no association between *ACTB* intensity and related prognostic factors. Furthermore, no association was observed between membranous expression of *ACTB* and survival rate, tumor grade, or tumor invasiveness. As shown in Fig. 6F, the survival analysis showed no association between the expression of *ACTB* and overall survival time.

### In silico analysis

**Fig. 7:**
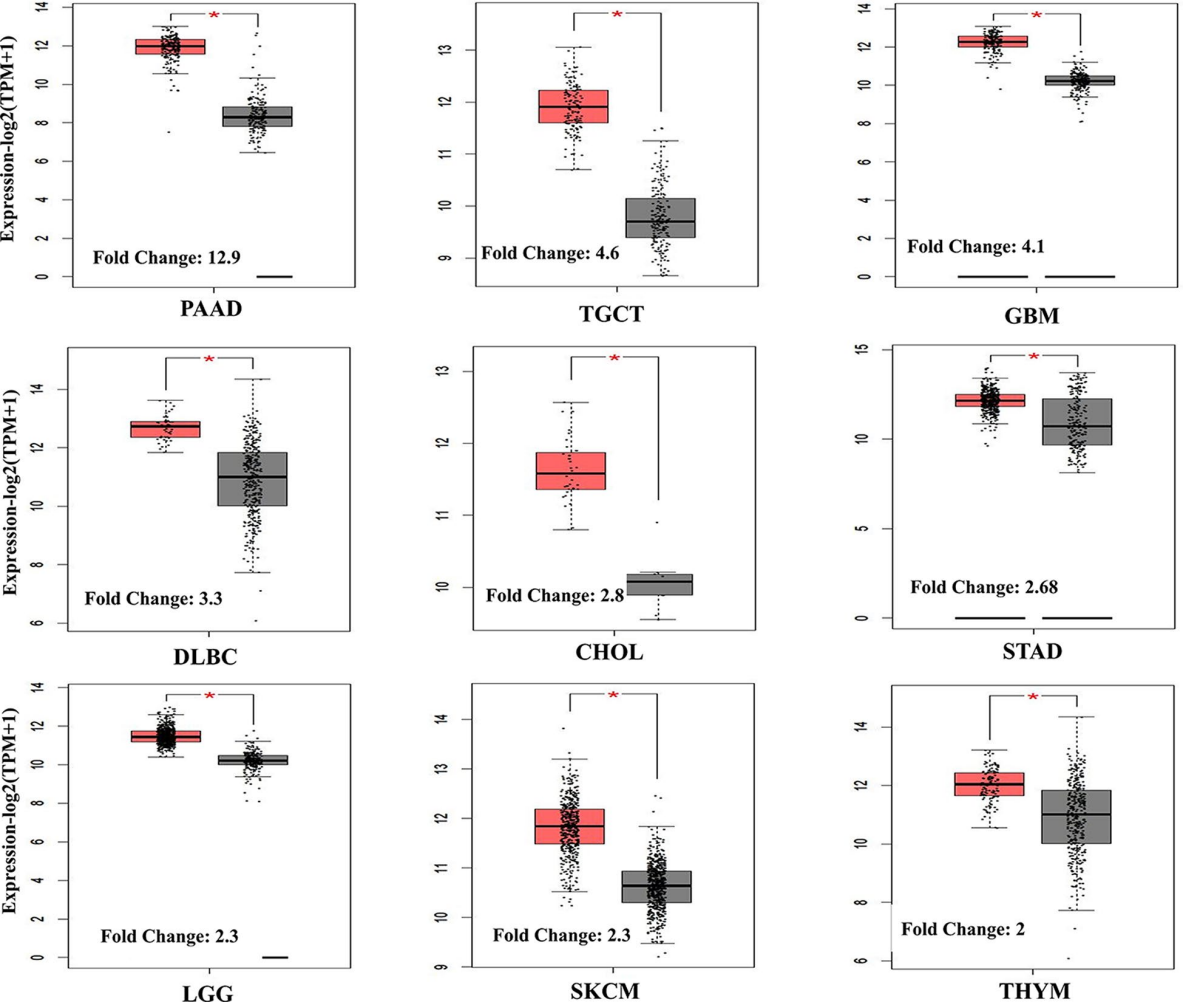
Differential expression of *ACTB* in various cancer tissues. There was a significant difference between the expression of *ACTB* in healthy and tumor tissue in 9 cancers (among 31 cancers), and the maximum difference was seen in pancreatic adenocarcinoma (PAAD; fold change = 12.9), testicular germ cell tumors (TGCT; fold change = 4.6), and glioblastoma multiform (GBM; fold change = 4.1). In silico data were obtained from the GEPIA2 database

**Fig. 8:**
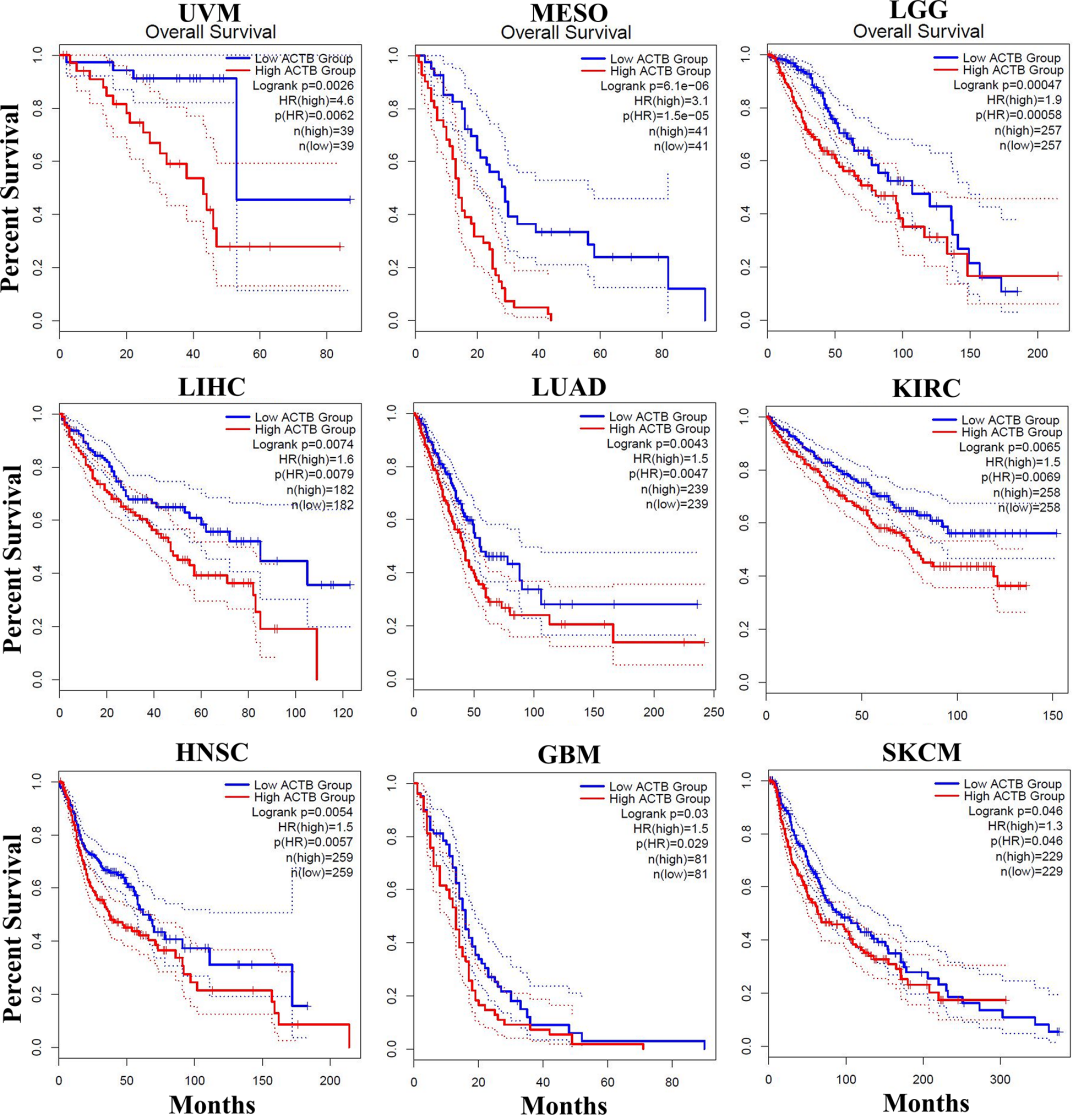
The overall survival time of patients with low or high expression. The expression of *ACTB* was associated with survival time in various cancers, such as GBM, head and neck squamous cell carcinoma (HNSC), kidney renal clear cell carcinoma (KIRC), LGG (brain lower grade glioma), liver hepatocellular carcinoma (LIHC), lung adenocarcinoma (LUAD), mesothelioma (MESO), SKCM (skin cutaneous melanoma), and uveal melanoma (UVM)

**Fig. 9:**
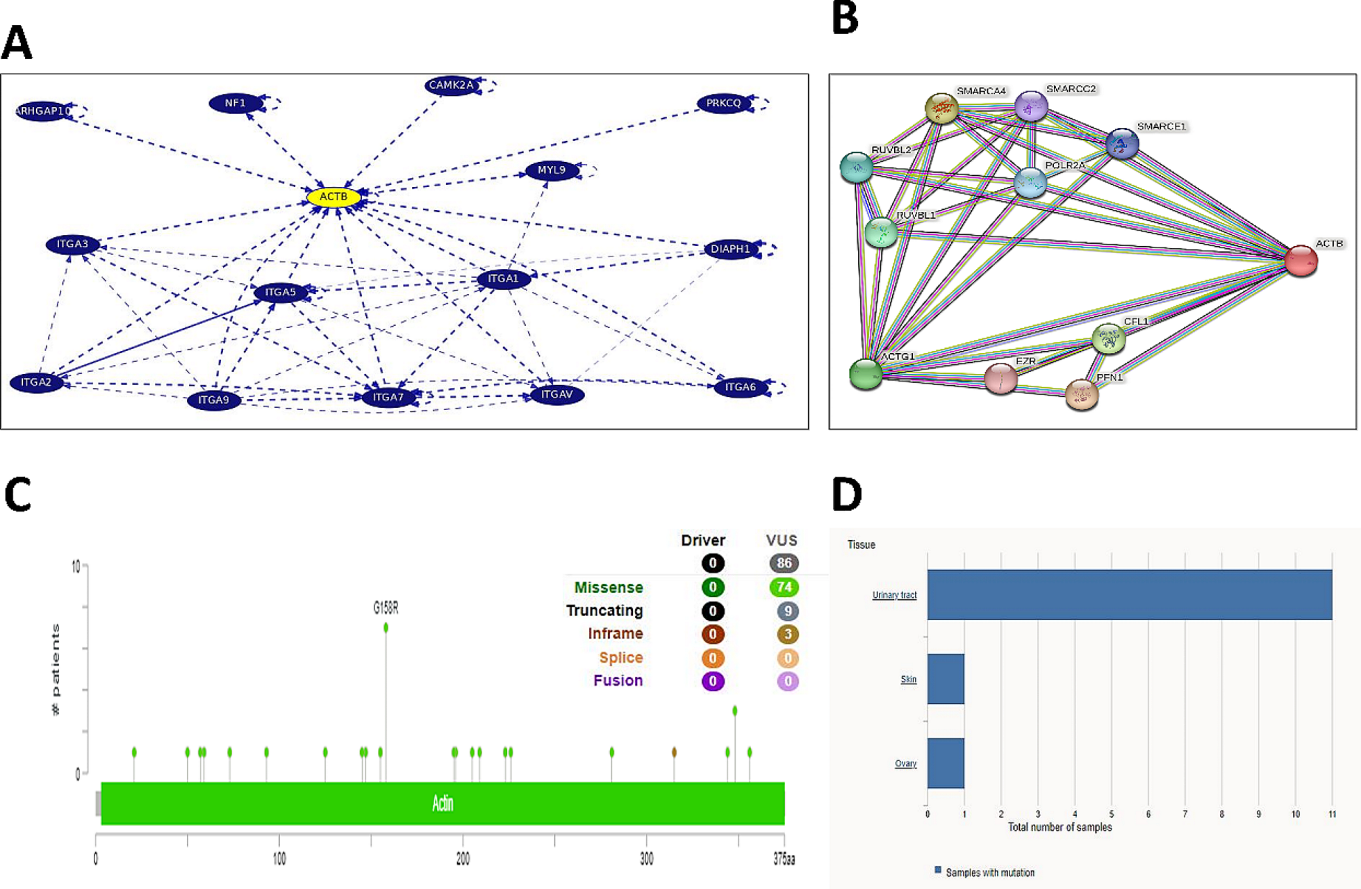
Interaction of *ACTB* with other genes **(A)** and proteins **(B).** The interaction network of *ACTB* with other proteins and genes was obtained from STRING and UCSC servers, respectively. Additionally, *ACTB* mutations in patients with BLCA (**C**) and the tissue distribution of the prevalent mutation (p.G158R) are shown (**D**)

Consistent with our results, bioinformatics analysis revealed that the expression of *ACTB* between the tumor and healthy tissues was not significantly different (Fig. 6A), and *ACTB* was not associated with survival rate in BLCA (Fig. 6E). However, our study did not show a significant difference, and higher expression of *ACTB* was observed in stage IV compared with stages II and III of BLCA in the GEPIA2 database (Fig. 6C). The significant correlations between *ACTB* and similar genes, overexpressed genes and BLCA biomarkers are summarized in Table 2. According to the profile of expression patterns, GEPIA2 proposed 10 genes as similar genes to *ACTB* in BLCA. Among them, the role of PTRF, ARPC1B, FLNA, and RHOC in BLCA pathogenesis was elucidated by previous studies [18–21]. *ACTB* also showed a weak to moderate correlation with BLCA tumor-associated antigens and overexpressed genes. As shown in Fig. 7, there was a significant difference between the expression of *ACTB* in healthy and tumor tissues in 9 cancers (among 31 cancers). The maximum differences were seen in pancreatic adenocarcinoma (PAAD; fold change = 12.9), testicular germ cell tumors (TGCT; fold change = 4.6), and glioblastoma multiforme (GBM; fold change = 4.1). Furthermore, the expression of *ACTB* was significantly associated with survival time in various cancers, such as GBM, head and neck squamous cell carcinoma (HNSC), kidney renal clear cell carcinoma (KIRC), LGG (brain lower grade glioma), liver hepatocellular carcinoma (LIHC), lung adenocarcinoma (LUAD), mesothelioma (MESO), SKCM (skin cutaneous melanoma), and uveal melanoma (UVM). Similarly, lower expression of *ACTB* has been associated with improved survival rates (Fig. 8). The interactions of *ACTB* with other genes (ITGA1, ITGA2, ITGA3, ITGA5, ITGA6, ITGA7, ITGA9, ITGAV, DIAPH1, MYL9, NF1, ARHGAP10, CAMK2A, and PRKCQ) and proteins, including CFL1, PFN1, EZR, SMARCE1, SMARCC2, POLR2A, SMARCA4, RUVBL2, RUVBL1, and ACTG1, are shown in Fig. 9. They act as intermediaries in the regulation of cell morphology, cytoskeleton structure, cell proliferation, chromatin remodeling, cell-to-cell adhesion, migration, and metastasis. In *ACTB* of patients with BLCA, 86 mutations were found, and all of them are variants of unknown significance (VUS), according to the cBioPortal database. They included 74 missense mutations, 9 truncate mutations, and 3 in-frame mutations. p.G158R (c.472G > A) is the most prevalent one that is the missense mutation type (Fig. 9C). Tissue distribution in the COSMIC database also showed the highest frequency of this mutation in the urinary tract (Fig. 9D).

**Table 2:** The correlation between *ACTB* and similar genes, over-expressed genes and BLCA biomarkers was obtained from the GEPIA2 database. The significant correlation was listed. The strength of relationship was categorized into three levels: weak (*R* = 0.0 to 0.29), moderate (*R* = 0.3 to 0.69), and strong (0.7 to 1)

Gene Symbol	*P*-value	Pearson correlation coefficient (*R*)	Correlation Statues
**Over-expressed Gene**			
UBE2C	0.0005	0.17	Weak
CDC20	< 0.0001	0.32	Moderate
MYBL2	< 0.0001	0.2	Weak
FAM83A	0.0014	0.16	Weak
TROAP	0.0055	0.14	Weak
KRT7	0.0052	-0.14	Weak
TK1	< 0.0001	0.3	Moderate
BIRC5	0.0006	0.17	Weak
AURKB	< 0.0001	0.23	Weak
TPX2	0.0004	0.17	Weak
FOXM1	< 0.0001	0.23	Weak
RRM2	0.001	0.16	Weak
PKMYT1	< 0.0001	0.34	Moderate
CCNB2	0.0033	0.15	Weak
KRT16	0.01	0.13	Weak
MMP11	< 0.0001	0.37	Moderate
**Tumor-associated Antigens**			
NUMA1	0.00014	-0.19	Weak
AURKA	< 0.0001	0.21	Weak
ALCAM	0.01	0.13	Weak
NNMT	< 0.0001	0.52	Moderate
KRT20	< 0.0001	0.28	Weak
APEX1	0.0034	-0.15	Weak
HAI1	0.0023	-0.15	Weak
CXCL8	0.017	0.12	Weak
FGFR3	< 0.0001	-0.28	Weak
APOE	0.0017	0.16	Weak
SDC1	< 0.0001	-0.27	Weak
SERPINA1	< 0.0001	0.31	Moderate
SERPINE1	< 0.0001	0.35	Moderate
P3H4	< 0.0001	0.36	Moderate
AP2S1	< 0.0001	0.49	Moderate
COL1A1	< 0.0001	0.34	Moderate
**Similar Gene**			
PPP1R18	< 0.0001	0.7	Strong
PTRF	< 0.0001	0.67	Moderate
RHOG	< 0.0001	0.63	Moderate
ARPC1B	< 0.0001	0.62	Moderate
RRAS	< 0.0001	0.61	Moderate
FHL3	< 0.0001	0.61	Moderate
FLNA	< 0.0001	0.61	Moderate
ACOT9	< 0.0001	0.61	Moderate
PTGIR	< 0.0001	0.6	Moderate
RHOC	< 0.0001	0.6	Moderate

## Discussion

The main goal of the present research was to develop and characterize monoclonal antibodies against the newly established BLCA cell line (JAM-ICR). In this connection, first, a hybridoma library against the JAM-ICR cell line was generated, and clones with high affinity for the JAM-ICR cell and low reactivity with normal cells were selected. Among several clones, the target of 6D6 mAb was characterized through IP, SDS‒PAGE, western blotting, and mass spectrometry analysis and determined to be *ACTB*.

The cytoskeleton of a eukaryote is composed of microfilaments, microtubules, and intermediate filaments, and the main component of microfilaments is actin with a molecular weight of 42 kD [22]. Among various actin isoforms, *ACTB* plays essential roles in proliferation, motility, integrity, structure, intracellular trafficking, endocytosis, chromatin remodeling, DNA repair, and regulation of transcription [23]. Several studies have reported that dysregulation of this protein is associated with the pathogenesis of several diseases. Differences in the expression of *ACTB* between healthy individuals and patients with asthma [24], Alzheimer’s disease [25], and congenital heart disease (CHD) have been reported [26].

*ACTB* isoforms are almost ubiquitously seen in all types of cells and accordingly are commonly used as internal controls to normalize the expression of other molecules [27]. In contrast, an increasing collection of research has recently proven that this protein varies in response to different situations and stimulations [28]. The expression of *ACTB* is influenced by several conditions, including hypoxia, hyperglycemia, infection, hydrocortisone, 17β-estradiol, angiotensin II, tumor necrosis factor-α, and sex-dependent hormones [29, 30]. In parallel, our study showed a differential expression level of *ACTB* on the surface of various cancer cell lines. Flow cytometry analysis showed strong, moderate and weak interactions of 6D6 with different cell lines. While 6D6 was bound to epithelial cells, it had little to no reactivity with SCs, MSCs, endothelial cells, or lymphocytes. Similarly, Lima et al. reported that *ACTB* is the most unstable gene under hypoxic conditions among referral genes in BLCA cells. Furthermore, the comparison of *ACTB* expression in resting and activated T cells revealed significant changes in the different statuses of lymphocytes [31]. Additionally, variations in *ACTB* expression were also reported during the differentiation of growing vessels and epithelial cells [32, 33]. Our findings, along with these studies, collectively indicated that using *ACTB* as a constitutive internal control is challenging.

Differential expression of *ACTB* was also observed in various cancers [28]. The upregulation of the *ACTB* gene in a rat hepatoma model compared to healthy controls was reported by Chang et al. [34]. This upregulation was also found in gastric cancer and was correlated with tumor grades [35]. In another study, the expression level of *ACTB* was evaluated in K562 cells, normal leukocytes and leukocytes of different malignancies. The expression level was different in this assessment depending on the origin and type of cells [36]. Similarly, to find a new biomarker for esophageal squamous cell carcinoma using two-dimensional gel electrophoresis (2-DE), Liu et al. found significantly increased expression of *ACTB* in tumor tissue compared to adjacent normal tissues [37]. Goidin et al. evaluated *ACTB* gene expression in two subpopulations of melanoma cells (1C8 and T1C3) derived from the tumor of one patient. Analysis of cDNA arrays showed upregulation of *ACTB* in invasive T1C3 melanoma cells compared to that in noninvasive 1C8 cells [38]. Our in vitro evaluation also revealed that *ACTB* has heterogeneous expression intensity in healthy and tumor bladder cells, but this difference was not significant. Furthermore, based on in silico evaluation, higher expression of *ACTB* was observed in PAAD, TGCT, GBM, lymphoid neoplasms diffuse large B-cell lymphoma (DLBC), cholangio carcinoma (CHOL), stomach adenocarcinoma (STAD), LGG, SKCM, and thymoma (THYM) compared to healthy related tissues. Due to the lack of certain biomarkers in most of these malignancies, *ACTB* may be a candidate to be used for diagnosis following further investigations. Although the exact mechanism of *ACTB* in tumorigenicity is less known, numerous studies have reported that *ACTB* may be involved in migration, invasion, and metastasis of cancer cells [39]. In hepatocellular carcinoma (HCC), two to three-fold increase in the expression of *ACTB* was indicated in the advanced stage of HCC [40]. Consistent with these findings, our experimental data indicated that *ACTB* expression was upregulated in stage III/IV compared to earlier stages. Information from the GEPIA2 database provided additional support for this finding (Fig. 6). Thus, this suggests the possible involvement of *ACTB* in the tumorigenicity of bladder cancer. However, further studies are needed to elucidate this function.

According to the GEPIA2 database, higher expression of *ACTB* was associated with lower survival times in several cancers, including GBM, HNSC, KIRC, LGG, LIHC, LUAD, MESO, SKCM, and UVM. The Human Protein Atlas (HPA) database (https://www.proteinatlas.org/) mentioned *ACTB* as an unfavorable marker just in HNSC and KIRC with a 0.001 significance level. *ACTB* may also serve as a prognostic indicator in the management of these malignancies. Thus, *ACTB* can be utilized for both diagnosis and prognosis in cancers such as GBM, SKCM, and LGG.

According to the HPA database, the intercellular and membranous expression of *ACTB* has been predicted. Consistently, our IHC staining revealed that the 6D6 clone could stain *ACTB* in the cytoplasm and even on the membrane of cells. However, Schevzov et al. found that overexpression of *ACTB* in myoblast cytoarchitecture is accompanied by an increased level of this protein on the surface of cells, whereas this upregulation was not seen in gamma-actin [41]. Overexpression of *ACTB* is also observed in an invasive sarcoma cell line that redistributes and concentrates on the tips of pseudopodia, which drive cell extension and facilitate tumor cell invasion [42]. Popow et al., following isolating an invasive form of hepatoma, Morris 5123, from paternal cells, observed a significantly increased level of *ACTB*, which mostly accumulated in the submembrane [43]. Nowak et al. showed the correlation between the expression level of *ACTB* and the state of actin polymerization with the metastatic potential of colon adenocarcinoma cells. They found overexpression of *ACTB* in EB3, a highly motile cell, with localization in the cortical ring beneath the cellular membrane [44]. Thus, different patterns of *ACTB* (membranous and cytoplasmic) may also be due to the motility and invasiveness features of cells. In our study, 66.7% of cells with membranous expression had high expression of *ACTB*, but this difference was not significant. Furthermore, no association was observed between membrane staining patterns of *ACTB* and prognostic factors.

Based on the GEPIA2 database, *ACTB* has a weak to moderate correlation with 16 overexpressed genes (from the first 25 genes) and tumor-associated genes in bladder cancer. Among similar genes, PTRF and ARPC1B were proposed as biomarkers for the prognosis and survival of BLCA [18, 19]. Moreover, FLNA regulates autophagy, and its overexpression reduces the migration and invasion of bladder tumors [20]. Furthermore, a higher level of RHOC was reported in muscle-invasive tumors than in superficially invasive tumors, which were associated with worse survival [21]. There is a prognostic value for the integrin α (ITGA) subfamily genes in BLCA that interact with *ACTB* [45]. Therefore, *ACTB* could play a role in the etiology and pathology of BLCA; however, its role needs to be more clarified.

## Conclusion

In this study, a hybridoma library against the newly established JAM-ICR cell line was generated, and a 6D6 clone with high affinity was selected. Following multiple steps of characterization, the target of 6D6 mAb was identified as *ACTB*. Flow cytometry staining showed significant different expression levels of *ACTB* (from weak to strong) on various cell lines. IHC staining showed both cytoplasmic and membranous expression patterns of *ACTB* on epithelial cells. Additionally, 6D6 could differentiate between epithelial cells and normal leukocytes and MSCs. 6D6 bound strongly to epithelial cells while showing weak to no reactivity to SMCs, endothelial cells, and SCs. No differences were found between tumor epithelial cells and adjacent normal epithelial cells or benign tumor tissue (von Brunn nests). Moreover, no relationship was seen between *ACTB* expression and survival rate or other prognostic factors. In silico evaluation further confirmed our in vitro results, indicating a significant relationship between *ACTB* and BLCA overexpressed genes or biomarkers. Despite the lack of a significant relationship, it appears that *ACTB* plays an indirect role in the pathogenesis of BLCA. Additionally, the differential expression of *ACTB* on tumor and healthy tissue as well as its correlation with survival time in a number of cancers may be indicative of this marker’s usefulness in prognosticating or diagnosing cancer.

### Electronic supplementary material

Below is the link to the electronic supplementary material.

Supplementary Material 1

Supplementary Material 2

Supplementary Material 3

Supplementary Material 4

## Data Availability

Data sharing is not applicable to this article as no datasets were generated or analysed during the current study.

## Declarations

The authors confirm that all methods were carried out in accordance with relevant guidelines and regulations in Ethics declaration section.

## Abbreviations

mAbs: Monoclonal antibodies
BLCA: Bladder cancer
*ACTB*: Beta-actin
HAT: Hypoxanthine-aminopterin-thymidine()
HT: Hypoxanthine-thymidine
MSCs: Adipose-derived mesenchymal stem cells
BSA: Bovine serum albumin
TMB: Tetramethylbenzidine
IP: Immunoprecipitation
DSS: Disuccinimidyl suberate
IHC: Immunohistochemistry
gepia2: Gene Expression Profiling Interactive Analysis-2
COSMIC: Catalog of Somatic Mutations in Cancer
UCSC: University of California Santa Cruz
SCs: Stromal cells
SMCs: Smooth muscle cells
PAAD: Pancreatic adenocarcinoma
TGCT: Testicular germ cell tumors
GBM: Glioblastoma multiforme
HNSC: Head and neck squamous cell carcinoma
KIRC: Kidney renal clear cell carcinoma
LGG: Brain lower grade glioma
LIHC: Liver hepatocellular carcinoma
LUAD: Lung adenocarcinoma
MESO: Mesothelioma
SKCM: Skin cutaneous melanoma
UVM: Uveal melanoma
VUS: Unknown significance
CHD: Congenital heart disease
DLBC: Diffuse large B-cell lymphoma
CHOL: Cholangio carcinoma
STAD: Stomach adenocarcinoma
THYM: Thymoma
HPA: Human Protein Atlas
ITGA: Integrin α
