# Supplementary figures and images for: Exploring heterogeneous expression of beta-actin (ACTB) in bladder cancer by producing a monoclonal antibody 6D6
Source: BMC Urol. 2024 Jun 12;24:124. doi: 10.1186/s12894-024-01489-6 (PMC11167769; doi:10.1186/s12894-024-01489-6)

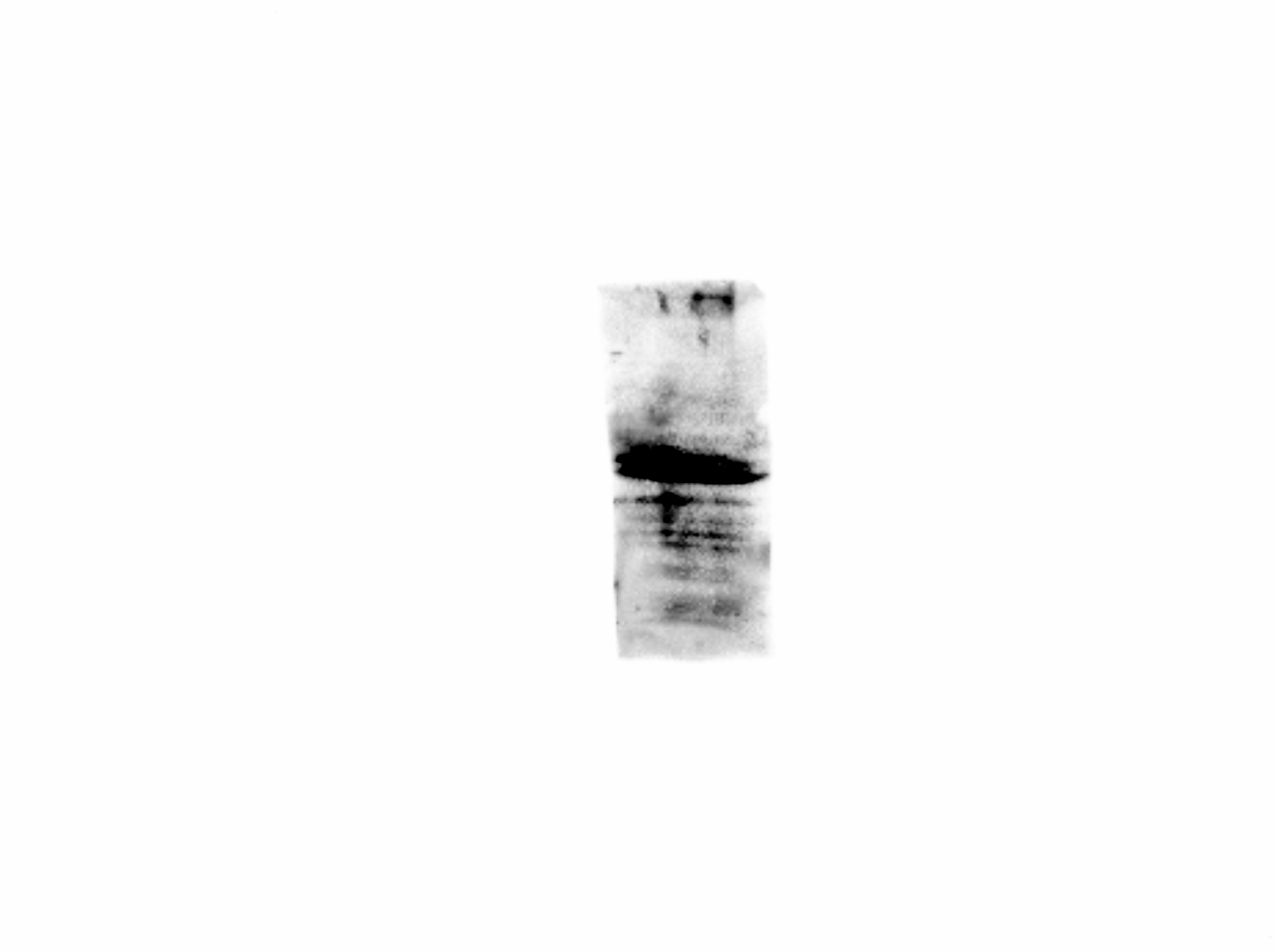

Supplement: Supplementary file 1 — Supplementary Material 1 [file 12894_2024_1489_MOESM1_ESM.png]

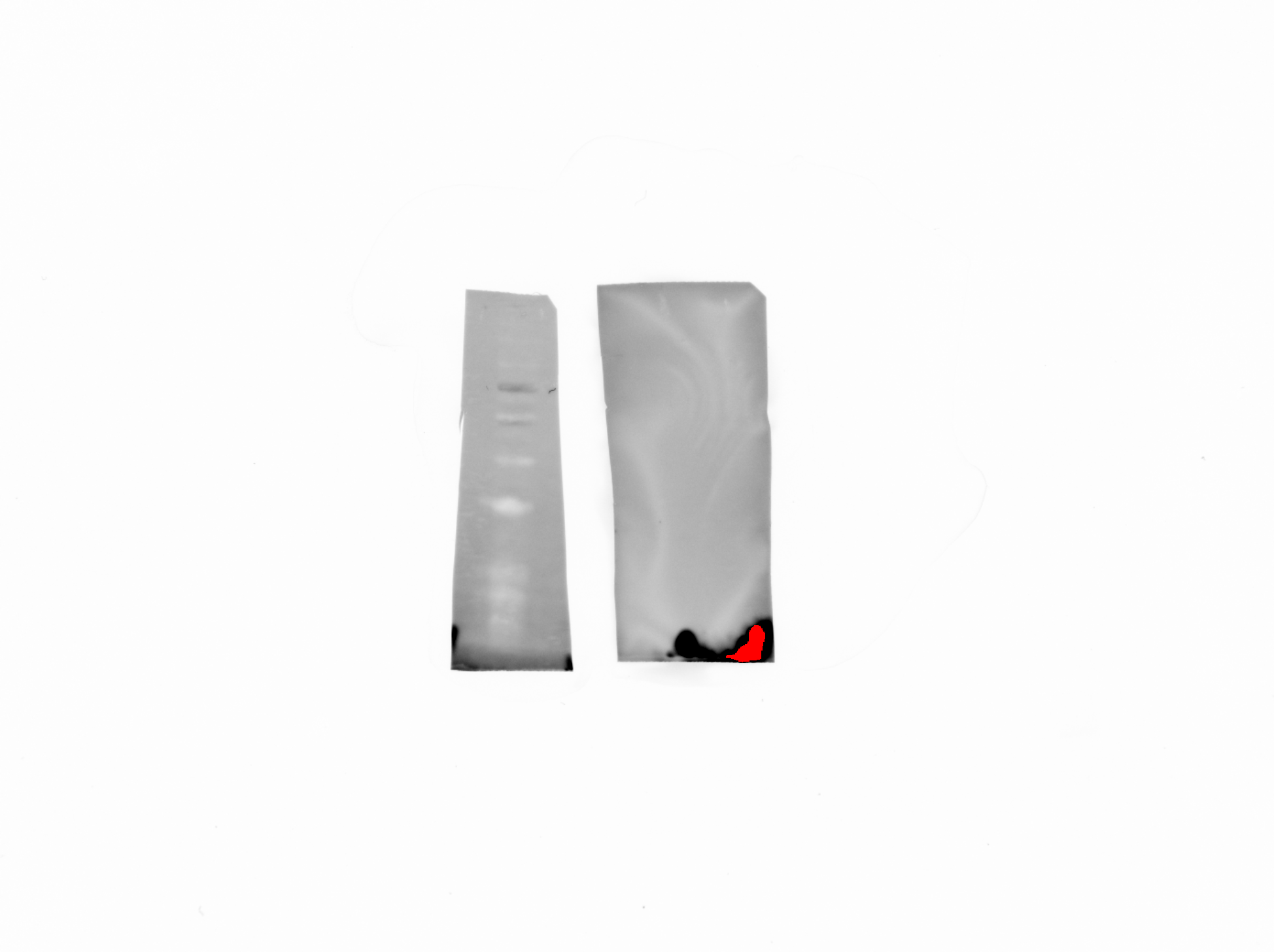

Supplement: Supplementary file 2 — Supplementary Material 2 [file 12894_2024_1489_MOESM2_ESM.png]

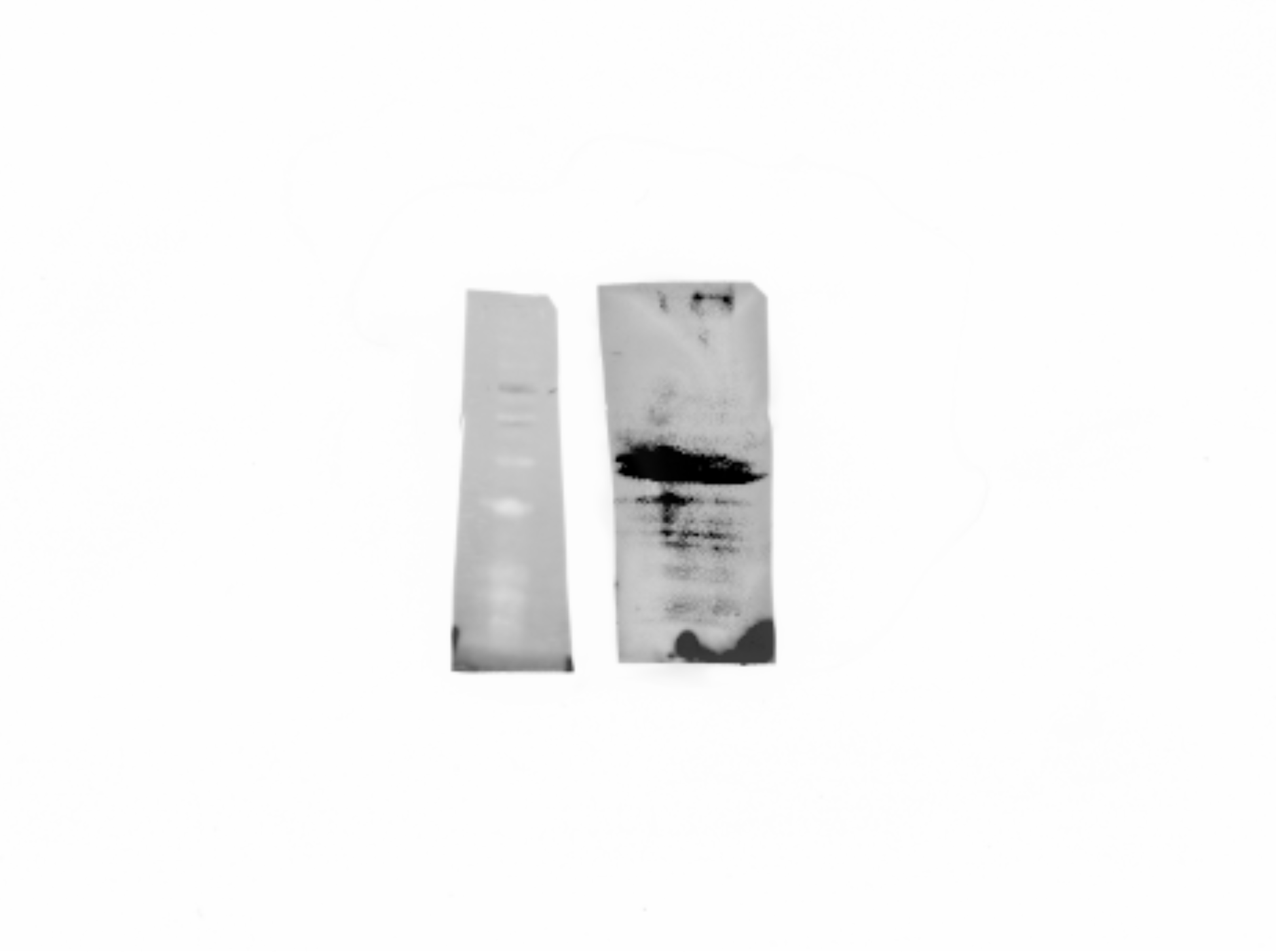

Supplement: Supplementary file 3 — Supplementary Material 3 [file 12894_2024_1489_MOESM3_ESM.png]
