# Supplementary material for: Exploring heterogeneous expression of beta-actin (ACTB) in bladder cancer by producing a monoclonal antibody 6D6
Source: BMC Urol. 2024 Jun 12;24:124. doi: 10.1186/s12894-024-01489-6 (PMC11167769; doi:10.1186/s12894-024-01489-6)
Supplement: Supplementary file 4 — Supplementary Material 4 [file 12894_2024_1489_MOESM4_ESM.docx]

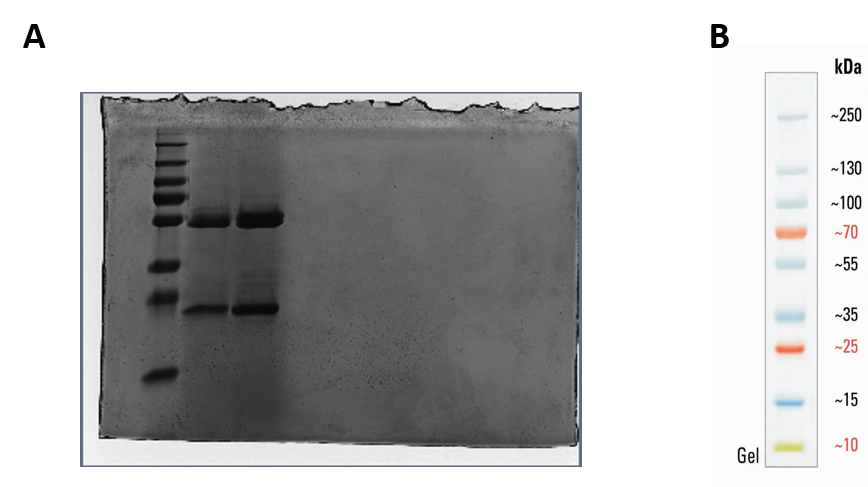


**Figure S1- Characterization of the purified mAb.** 6D6 with two concentrations (10 and 20 µg) were loaded on a 12.5% polyacrylamide gel under reduced conditions. After staining the gel with colloidal Coomassie Brilliant Blue G-250 and destaining, the image of the gel was captured with GS-800 Calibrated Densitometer (A; Bio-Rad). MW was determined using a protein marker (B; Thermo Scientific, 10 to 250 kDa).


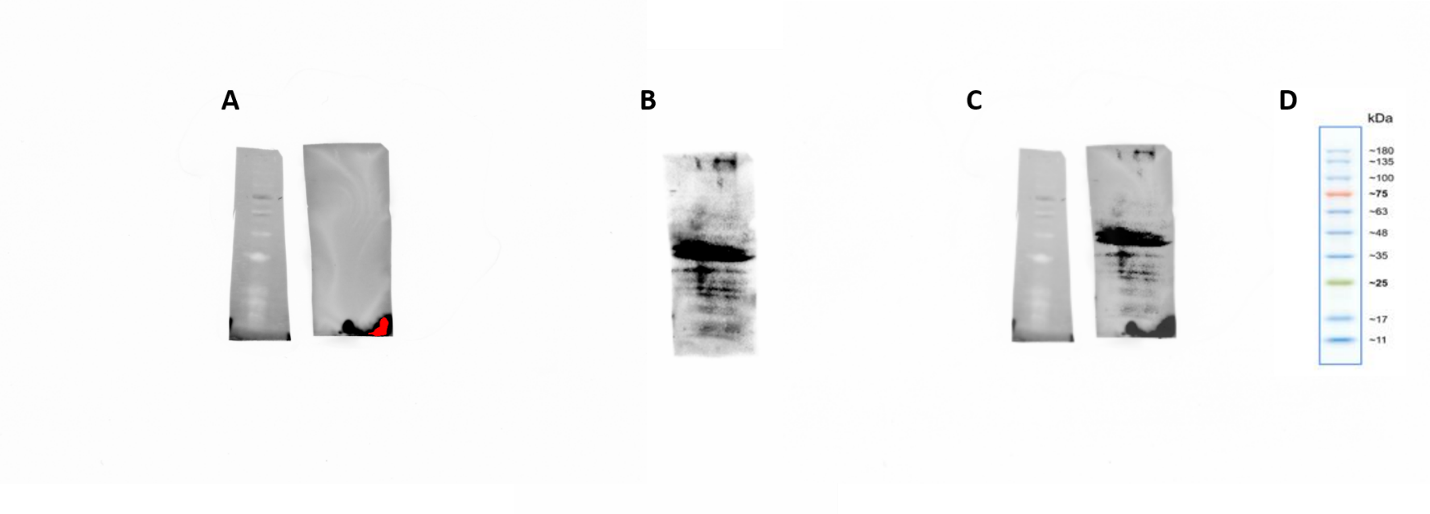


**Figure S2- Characterization of the target antigen of 6D6 mAb.** After transferring, membranes were cut before hybridization with the antibody. Images were captured with stain-free blot (A) and chemi (B) settings and then merged with Image Lab software version 6.1.0 (C, Bio-Rad). The MW of the target antigen was obtained using a pre-stained marker (D; Sinaclon, Cat.No. SL7011(PR911654)).


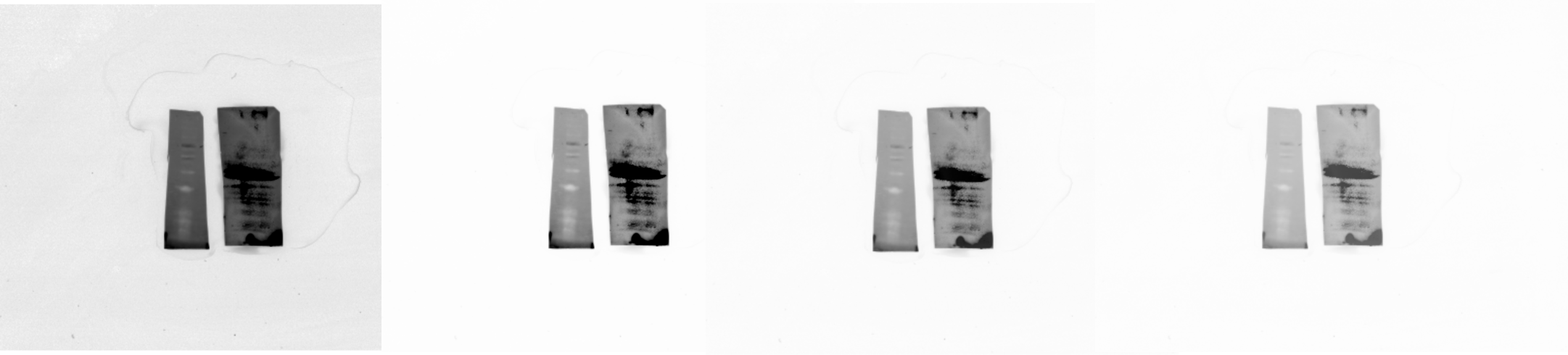


**Figure S3- Multiple contrast percentages of the merged image (Figure 2B) with** **Image Lab software.**
